# Improving Clinical Generalization of Pressure Ulcer Stage Classification Through Saliency-Guided Data Augmentation

**DOI:** 10.3390/diagnostics15232951

**Published:** 2025-11-21

**Authors:** Jun-Woo Choi, Won Lo Rhee, Dong-Hun Han, Minsoo Kang

**Affiliations:** 1 Department of Medical Artificial Intelligence, Eulji University, Seongnam 13135, Republic of Korea; chlwnsdn456@naver.com (J.-W.C.); d555v@naver.com (D.-H.H.); 2 NASCALAB, Seongnam 13105, Republic of Korea; nascalab@gmail.com; 3 Department of Bigdata Medical Convergence, Eulji University, Seongnam 13135, Republic of Korea

**Keywords:** pressure ulcer, classification, curriculum learning, generalization, YOLOv7, saliency map, data augmentation, domain shift

## Abstract

**Background/Objectives**: This study demonstrates improved generalization in pressure-ulcer stage classification. In medical imaging, training data are often scarce and disease specific. For skin conditions such as pressure ulcers, variation in camera to subject distance, resolution, illumination, and viewpoint across photographers reduces accuracy in clinical use. **Methods**: We developed a YOLOv7-based pressure ulcer stage classification model by employing a two-phase training strategy. Phase 1 was trained on the full dataset stratified by pressure-ulcer stage. Phase 2 was trained in saliency-guided images augmented with clinically plausible noise, including healing areas and white keratin. The added dataset comprised 296 images obtained by randomly sampling 30% from stages 1 through 3 of the full dataset. **Results**: The accuracy of the 38 newly acquired hospital images increased from 75% in Phase 1 to 89% in Phase 2. Five-fold cross-validation demonstrated stable performance (mAP@0.5: 86.20% ± 2.28%), confirming reproducibility. This exceeds by more than five percentage points the performance reported for pressure-ulcer staging models in prior studies conducted in clinical deployment settings. **Conclusions**: These findings suggest that curriculum learning combined with noise-enriched augmentation can improve generalization in clinical environments. Our results demonstrate that clinically informed data augmentation is a key factor in enhancing the model’s clinical generalization. Accordingly, the proposed approach provides a practical path to enhancing clinical usability in data-limited medical imaging.

## 1. Introduction

Machine learning and deep learning are widely used in medical imaging. Photographs acquired in clinical practice can differ markedly owing to minor variations in camera to subject distance and angle, illumination, and color balance. Reflections, shadows, and sensor noise are also common. These factors cause brightness and color contrast to vary across images of the same lesion, creating a distribution gap between internal validation data and the data encountered in hospital settings. Under these conditions, the available data is limited and imbalanced, which hinders model generalization. To achieve strong generalization, large datasets are typically required to avoid overfitting [1]. Models trained only in clean images can be vulnerable to noise artifacts present in real clinical environments. Artificial noise injection has been used as a data augmentation method to approximate real conditions [2]. Data augmentation increases diversity without collecting new samples and can mitigate overfitting and class imbalance [3]. Numerous augmentation methods have been proposed for this purpose [4]. However, augmentation based solely on simple image transformations tends to produce many similar samples and fails to capture the variations encountered in clinical care. Because augmentation can yield substantial gains in classification performance, it is reasonable to adopt a targeted strategy that bolsters clinically underperforming classes to add the necessary diversity [4]. Recent studies explicitly call for stronger generalization beyond internal test sets and for clinical validation [5]. Explainability is also repeatedly cited as a core requirement in safety critical workflows [5].

Images of stage 1 through 3 pressure ulcers contain subtle visual cues, and judgment consistency varies with clinician experience and training, which reduces discriminability between stages [6]. Augmentations that erase parts of the input or overwrite regions with random pixel values reduce bias toward easy cues and promote robustness to clinical distractors such as occlusions, highlights, and tape. In this study, augmentation was manually crafted to match real distractors in clinical photographs and was practical when implementation must be simple and data are scarce [7]. Saliency maps indicate which regions a model uses as evidence for its decisions and are widely used in medical imaging [8]. Using a saliency guided approach, we verified that the model focused on clinically valid lesion regions and, incorporating hospital feedback on pressure ulcer image characteristics that the model failed to detect, designed targeted noise and occlusion augmentations. Following additional training, we evaluated both internal validation, accuracy and performance in hospital to verify the results.

### Related Works

Model generalization depends on the training strategy. Common approaches include random sampling-based training, data augmentation, and hard example mining. Random sampling-based training can be inefficient and unstable in early learning [9], while focusing on the most difficult samples can induce overfitting or training instability [10]. To improve generalization efficiently, we adopted curriculum learning. In this approach, the model first learns from data in which lesions are clearly visible (lower difficulty) and then progressively tackles more difficult concepts [11]. Empirically, studies in chest X ray lung disease classification and in histopathology have shown that starting with samples that exhibit high inter-rater agreement among specialists, followed by training on difficult and complex lesion patterns, improves generalization [12,13].

The You Only Look Once (YOLO) family of object detection models demonstrates strong performance on rule-based tests and standardized datasets. However, performance often drops in real-world clinical settings, primarily because training data differ from clinical data. Wang and colleagues modified YOLOv5 with multi-attention and, on their own test set, increased recall by 0.045 over YOLOv8 and by 0.027 over a two-stage R-CNN [14]. They reported that existing YOLO models miss low-contrast or small polyps. This illustrates that performance measured only on common benchmark datasets fails to capture the diversity of real environments. A study on endoscopy published in Sensors reported that specular reflection interferes with segmentation of cervical lesions, polyp detection in colonoscopy, and classification of laryngeal cancer [15]. The proposed technique for detecting and removing specular reflections outperformed other methods, achieving accuracy 0.98, F1 score 0.81, Dice 0.76, and Jaccard 0.82. The authors also noted that correcting image appearance improves downstream detection and segmentation. These observations indicate that clinical light sources, reflections, and exposure conditions can induce errors in YOLO-based detectors. In addition, Krishnapriya and Karuna combined YOLOv7 with GrabCut on the BR35H brain tumor dataset and achieved accuracy 0.99, recall 1.0, and Dice 0.91 [16]. Taken together, these results show that pairing detectors with preprocessing and postprocessing tailored to field conditions can substantially shift performance, suggesting that pipeline design is often more decisive than adopting the newest model. In other words, the newest YOLO variant is not inherently superior; the key is a field-tuned design.

Medical imaging datasets are typically small and potentially biased, and acquisition conditions are inconsistent; as a result, model generalization tends to degrade. Across studies on pressure-ulcer stage detection, a performance drop at Stage 2 has been consistently observed. Lau and colleagues augmented 190 original pressure-ulcer images to obtain 1278 training images and developed a YOLOv4 smartphone app; on a validation set of 144 images, specificity for general wound detection was 0.85, whereas stage-wise sensitivities were 0.73 for Stage 1, 0.37 for Stage 2, 0.76 for Stage 3, 0.70 for Stage 4, and 0.55 for unclassified cases, underscoring the difficulty of intermediate stages with subtle tone and texture cues [17]. Aldughayfiq et al. performed joint bounding-box detection and stage classification with YOLOv5, reporting mAP@0.5 of 0.76 and stage-wise accuracies for Stages 1 to 4 of 1.00, 0.17, 0.65, and 0.82, corroborating Stage 2 as a weakness [18]. That study also highlighted the need for precise region specification to match wound shape, inclusion of non-pressure-ulcer wounds, and careful choices such as lightweight models, emphasizing the importance of refined data and model design.

Subsequently, YOLOv8 introduced structural improvements such as separated heads and anchor-free prediction, which are favorable for irregular wound morphology and mobile applications. Tusar and colleagues built a clinician-validated, class-balanced dataset and, using YOLOv8s with AdamW, achieved mAP@0.5 of 0.84 and recall of 0.82; stage-wise accuracies for Stages 1 to 4 were 0.74, 0.76, 0.70, and 0.77, indicating improvement at Stage 2 relative to earlier YOLO variants [19]. From a deployment perspective, Chang and colleagues trained five YOLOv8 models on 2800 annotated images and found YOLOv8m to be the top performer, with an accuracy of 0.846, precision 0.897, recall 0.891, F1 score 0.894, and mAP@0.5 of 0.908. Through TorchScript optimization, they ported the model to a smartphone app with inference in about 3 s, and they discussed the benefits of anchor-free detection relative to Faster R-CNN, emphasizing wound-localization accuracy [20]. Collectively, these case studies highlight the vulnerability to specular highlights, noise, and viewpoint shifts and the need for targeted augmentation that injects realistic variation while preserving diagnostic cues.

For targeted data augmentation, this study employed noise-based augmentation tuned to lesion scale and texture, together with a saliency-guided approach that encourages editing away from clinically important regions. Bae et al. proposed an argumentation that mixes class-labeled High-Resolution Computed Tomography (HRCT) Region of Interests (ROI) with Perlin noise. By blending disease patches in HRCT using Perlin-noise masks, pixel-level classification accuracy increased compared with traditional augmentation. In the same CNN, ROI-level accuracy improved from 0.82 to 0.89, and whole-lung quantification accuracy improved from 0.45–0.49 to 0.49–0.55, emphasizing that when the mask scale is smaller than the target pattern, it can corrupt the relevant signal and degrade performance; thus, matching the design to disease size and texture is important [21]. A learning-to-augment strategy learns the type and intensity of injected noise via Bayesian optimization and pairs this with a denoising autoencoder to create semantics-preserving yet diverse views. A case study reported improved COVID-19 chest X-ray classification over prior LTA, with sensitivity 0.808, specificity 0.915, and F-measure 0.737 [22].

Because conventional cut-paste or grid-mixing can occlude key anatomy, saliency-aware transformations use heat maps such as Grad- Class Activation Map (CAM) and log-spectrum saliency to perform paste, erase, and emphasize operations around clinically important features. To overcome the limitation that rectangular masks can occlude anatomy, Hammoudi and colleagues proposed SuperpixelGridCut, SuperpixelGridMean, and SuperpixelGridMix. FocusAugMix integrates Grad-CAM, SaliencyMix, and multi-head attention on superpixels, and it consistently outperformed CutMix, GridMix, OccaMix, and saliency-only methods on a leukemia-cell dataset while steering augmentation away from important regions [23]. With VGG19, the PASCAL VOC score improved from 0.57 to 0.75 using SuperpixelGridMean or SuperpixelGridMix. Grad-CAM and attention blocks also support the explainability required in clinical workflows, allowing visualization of model focus and enabling augmentation to be tuned to disease-related morphology [23]. In related work, Mustaqim and colleagues integrated FocusAugMix with Grad-CAM, multi-head attention, and SaliencyMix on superpixels to encourage editing away from important regions, achieving accuracy up to 0.99 on acute lymphoblastic leukemia data and outperforming SaliencyMix, CutMix, and GridMix [24]. These results suggest that saliency and attention methods can preserve clinical cues while expanding diversity, even with small datasets.

This study proposes a two-phase, domain-tailored training schedule that combines saliency-guided protection with targeted clinical overlays. Saliency maps identify stage-defining regions to be preserved, while overlays emulate common bedside distractors, including gauze or tape edges, ointment residues and specular highlights. The overlays are applied selectively to samples from the most confusable stages to increase diversity without corrupting diagnostic cues. This staged schedule differs from prior augmentation-only or curriculum-only designs by coupling explainability-driven protection with distractor-specific augmentation tuned to pressure-ulcer staging.

## 2. Materials and Methods

### 2.1. Preparing Decubitus Stage Detection Model

Data for this study were obtained with approval from the Institutional Review Board of Daejeon Eulji University Hospital. After anonymization by physicians in the hospital’s QI office, images were categorized by the pressure ulcer stage; for each stage, photographs in which lesion morphology was clearly visible were selected. The dataset comprised 1312 images labeled into four classes (Stages 1 to 4). The distribution by stage is summarized in Table 1.

**Figure 1:**
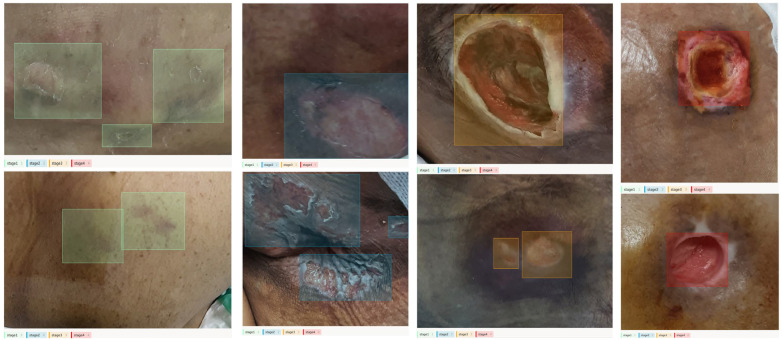
Data Labelling. The colored bounding boxes indicate the specific stages: green (Stage 1), blue (Stage 2), orange (Stage 3), and red (Stage 4).

This class imbalance reflects real-world ward patterns and day-to-day recording practices. Stage 2 injuries are the most commonly observed categories across acute-care datasets [25]. Stage 1 findings such as non-blanchable erythema are subtle and particularly difficult to visualize, especially in patients with darker skin tones, so they are under-imaged [26]. Severe Stage 4 injuries are relatively infrequent in contemporary acute-care cohorts [25]. Routine wound-care workflows, for example, imaging around dressing changes, also contribute to accumulating more photographs for Stage 2, whereas documentation is more selective in advanced cases [27]. Labeling was performed using the open-source tool Label Studio. As shown in Figure 1, the bounding boxes were limited to the representative lesion area that best captured the pathological characteristics of the corresponding stage.

We chose the YOLO family due to its lightweight architecture, strong generalization with limited data, and proven performance in medical imaging. Notably, Brüngel and Friedrich reported that YOLOv5 consistently outperformed DETR in a diabetic foot ulcer dataset under equal training conditions, especially when data were limited and clinical realism was required [28].

Several versions of the YOLO family have been released with varying architectural innovations and performance characteristics. While YOLOv8 offers an anchor-free, decoupled head and a streamlined C2f backbone [29], and YOLOv6 integrates industrial-grade optimizations such as task-aligned label assignment and RepVGG-based backbones [30], we selected YOLOv7 for its strong balance of accuracy, stability, and practical training behavior under limited data conditions. YOLOv7’s E-ELAN backbone and auxiliary head provide deep feature aggregation and robust supervision, which are particularly beneficial in detecting small or ambiguous wound boundaries. Comparative studies have shown that YOLOv7 frequently performs on par with or better than YOLOv5 and YOLOv8 in real-world or low-data domains [16], while maintaining architectural maturity and reproducibility desirable in clinical AI workflows.

### 2.2. Phase 1: Standard Augmentation Baseline

For pressure-ulcer stage detection, we used the YOLOv7 object-detection model. Although deep learning typically requires large, labeled datasets for training, transfer learning enables strong performance even with small datasets [31]. Accordingly, we initialized each model with pre-trained weights from the COCO dataset and then trained the models. All models used the same hyperparameters (lr0 = 0.01, lrf = 0.1, batch size 64, 200 epochs) and were trained on a workstation with an NVIDIA RTX 3090 GPU (NVIDIA, Santa Clara, CA, USA). Training was conducted with the PyTorch-based Ultralytics library (Version 8.3.100). We used Ultralytics default data augmentation with the following magnitudes adjusted: horizontal flip probability 0.5, vertical flip 0.0, rotation ±45°, translation 0.10, scale 0.90, shear 0.0, perspective 0.0, and HSV jitter h 0.015, s 0.70, v 0.40. Mosaic was set to 1.0 and thus applied to all images, MixUp to 0.2, and Copy-Paste variants were disabled. This recipe served as the standard augmentation baseline for comparison with the proposed Phase 2 strategy. The overall training procedure is summarized in Algorithm 1.
**Algorithm 1** Pseudocode of Pressure Ulcer Stage Classification Using YOLOv71:**function** CLASSIFICATION2:dataset ← LOADDATASET (Pressure_Ulcer_Image)3:  classes ← [Stage1, Stage2, Stage3, Stage4]4:  train_set, val_set ← SPLITDATASET (dataset, retio = 0.8)5:  models ← [TOLOv7]6:  **for** model do7:   TRAIN (model, train_set)8:   predictions ← PREDICT (model, val_set)9:   precision ← COMPUTEPRECISION (prediction, val_set.labels, classes)10:    recall ← COMPUTERECALL (prediction, val_set.labels, classes)11:   mAP@0.5 ← COMPUTEMAP (predictions, cal_set.labels, 0.5)12:   mAP@0.5:0.95 ← COMPUTEMAP (predictions, cal_set.labels, [0.5, 0.95])13:   STORERESULTS (precision, recall, mAP@0.5, mAP@0.5:0.95)14:   **end for**15:COMPARERESULTS16:**end function**

**Figure 2:**
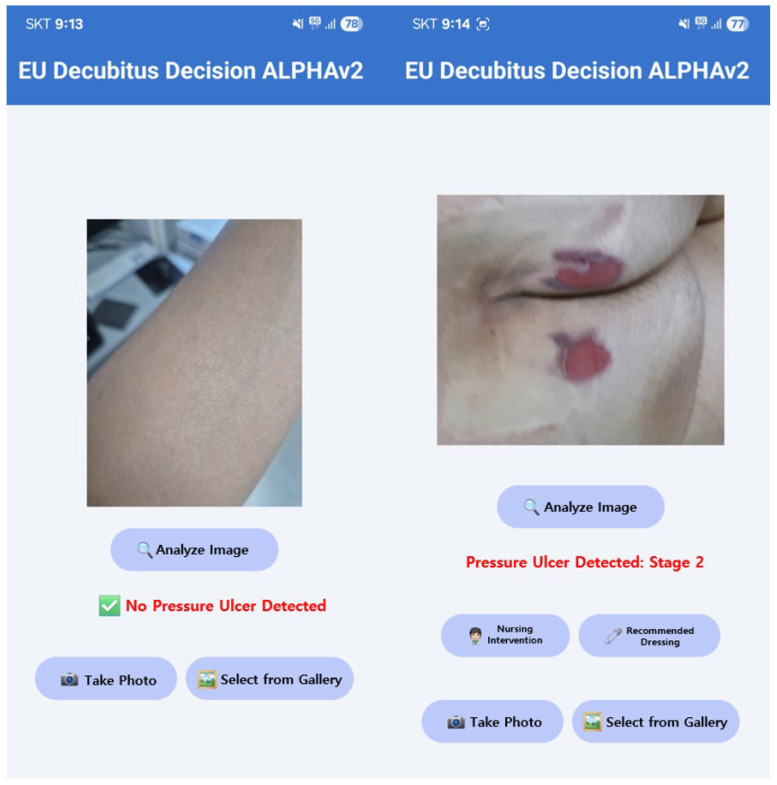
Pressure ulcer application.

Building on this work, we deployed the system as an application at Eulji University Hospital, as shown in Figure 2. The test photographs included an author’s arm and a patient’s pressure ulcer. The application provides pressure-ulcer detection, stage-specific treatment guidance, and database functions for image capture and storage.

### 2.3. Saliency Map for Pressure Ulcer Stage Prediction

A saliency map visualizes the image regions that influenced the model when predicting the pressure ulcer stage. Early methods indicate sensitivity by using the gradient of the input with respect to the class score [32], whereas Grad CAM uses the gradient of the class score with respect to intermediate feature maps to produce a heat map applicable to any CNN [33]. In this study, we used an activation-based CAM-style saliency map to identify the regions the model attends to when predicting the pressure ulcer stage, and we incorporated these findings into the design of data augmentation. The detailed process for generating the saliency map is presented in Algorithm 2.
**Algorithm 2** Pseudocode of Activation-based Saliency1:**function** SINGLE_LAYER_SALIENCY (image, model, selected_layer, size)2:  cache ← ATTACH_HOOKS_AND_RUN (model, image, selected_layer)3:  detections ← GET_DETECTIONS (cache)4: **for** bbox/laber overlay only5:  feature_map ← GET_FEATURE_MAP (cache, selected_layer)6:  a_single ← AVERAGE_CHANNELS (feature_map)7:  channel mean8:  a_single ← NORMALIZE_AND_RESIZE (a_single, size)9:  VISUALIZE (image, a_single, detections)10:  **return** a_single11: **end function**

**Figure 3:**
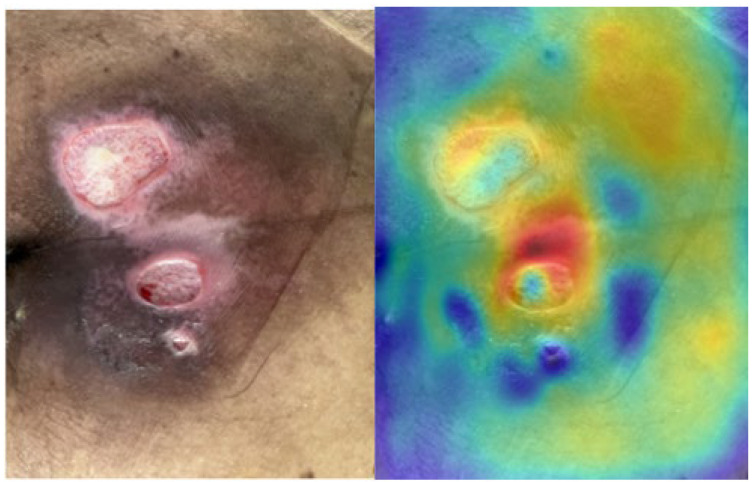
Example of Saliency map.

We installed forward hooks on all Conv2d layers to cache the feature maps of the later convolution blocks in a single forward pass [34], and from a selected layer, we computed a single-layer map by channel-wise averaging [35]. Let the selected convolutional layer be l and its feature map be $F_{l}$. The single-layer saliency map $A_{single}$ is given by Equation (1):

(1) $$A_{single}\left(x,y\right)=\frac{1}{C_{l}}\sum_{c=1}^{C_{l}}F_{l}\left(c,x,y\right)$$

where $F_{l}\left(c,x,y\right)$ denotes the activation at spatial location $\left(x,y\right)$ in channel c of layer l, and $C_{l}$ denotes the number of channels in layer l. To avoid contrast loss and improve interpretability, we used a single-layer map as the final saliency map rather than aggregating multiple layers. An example is shown in Figure 3.

The colors ranging from blue to red indicate an increasing model focus for pressure-ulcer stage determination. When designing the noise-based augmentations, we applied slight degradations to the red regions and their vicinity.

### 2.4. Phase 2: Saliency-Guided Clinical Overlays

**Figure 4:**
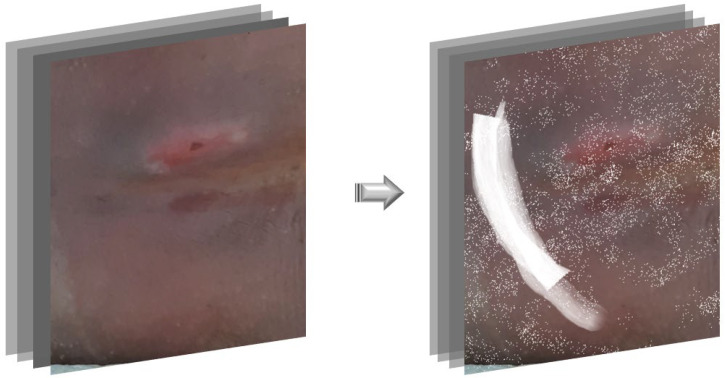
Example of white overlay data augmentation.

Our two-phase procedure is not a canonical curriculum that strictly partitions data by difficulty. Phase 2 partially overlaps with Phase 1 in image identities, and the cumulative number of unique images differs across phases because Phase 2 introduces saliency-guided overlays rather than entirely new patients. We therefore describe our approach as a staged training schedule with targeted augmentation. This data augmentation is a targeted strategy intended to address vulnerability in early stages where inter-stage boundary cues are subtle. In skin-wound images, thin linear structures can easily occlude lesions. Accordingly, training with synthetic hair and straight-line overlays improves invariance to hair occlusion [36]. In addition, wounds such as pressure ulcers, plastic dressings, ointments, and moisture-induced highlights and glare can confuse detection; in endoscopy, augmentation that artificially adds specular highlights has been shown to reduce false positives and increase robustness to field conditions [37,38]. Therefore, in this study, we used saliency maps as a visual guide. We manually overpainted noise onto a perilesional band while visually avoiding the high-saliency (stage-defining) regions thereby generating additional training samples. As shown in Figure 4, we overlaid white spray over and around the lesion and inserted white brush strokes sparingly along the lesion periphery. This mimics the particulate residues from dressings and ointments and the linear patterns at the edges of gauze and tape. Phase 2 retained the same base augmentation as the baseline and added saliency-guided, clinically realistic overlays around the lesion periphery while protecting stage-defining regions.

In medical imaging, mixing noise data at a proportion of 20% to 30% during training tends to significantly improve the discrimination and segmentation accuracy for new medical images [39,40]. Accordingly, the data composition consisted of a random 30% sample from the training set of each stage, excluding Stage 4, drawn from the total 1282 images. The 296 generated images were then randomly split while maintaining the training to validation ratio of 8:2.

To evaluate the stability and reproducibility of the Phase 2 augmentation strategy, we conducted stratified 5-fold cross-validation starting from the Phase 1 best model. The training dataset was partitioned into 5 folds with approximately equal size while maintaining the original class distribution within each fold. For each fold, the Phase 1 best model (best.pt) was used as the initialization, and additional training was performed for 100 epochs with a batch size of 64 using the Phase 2 augmentation settings described above. Model performance was evaluated on each validation fold using standard object detection metrics: Precision, Recall, mean Average Precision at IoU threshold 0.5 (mAP@0.5), and mAP at IoU thresholds from 0.5 to 0.95 (mAP@0.5:0.95). These metrics were computed both for overall model performance and separately for each pressure ulcer stage.

## 3. Results

### 3.1. Phase 1 Training Results

**Figure 5:**
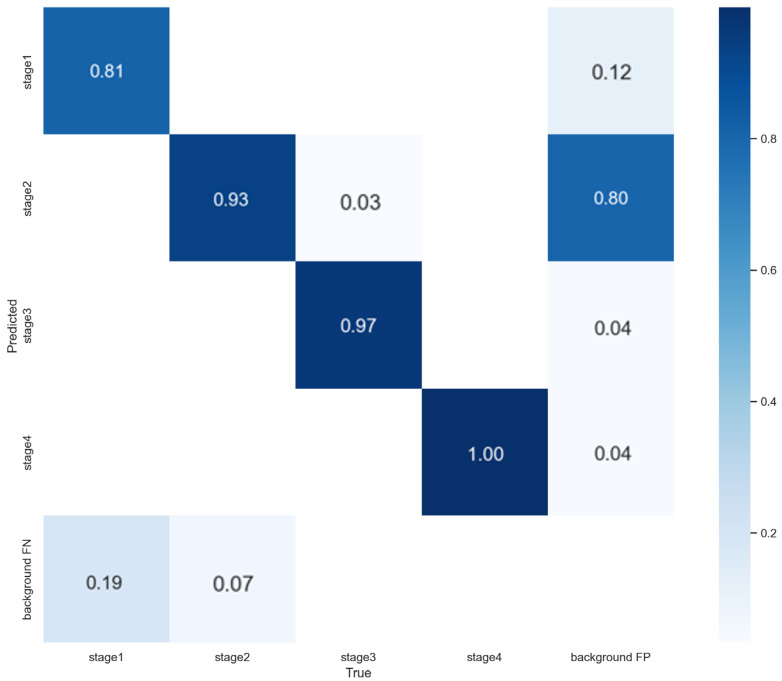
Confusion matrix of YOLOv7-based pressure ulcer stage classification model.

The Phase 1 model for pressure-ulcer stage classification was trained on 1282 pressure-ulcer images using YOLOv7. Figure 5 shows the stage-wise performance as a confusion matrix. Recall for Stage 2 was the lowest, and most errors occurred between adjacent stages. This pattern suggests that some false positives may arise in the localization step.

Across the pressure ulcer stages, the model achieved high classification accuracy; notably, the accuracy for Stage 2, which nurses often find the most challenging, reached 0.93. For Stage 4, whose lesion characteristics were pronounced and readily identifiable, the model achieved 1.00. The application developed on this basis was deployed and tested for 3 months, and the nurses reported a field accuracy of approximately 0.80. In practice, misclassifications occurred particularly when distinguishing Stage 2 from Stage 3. During healing transitions, when a pressure ulcer improved from Stage 2 to Stage 1 or from Stage 3 to Stage 2, the model often failed to detect the lesion. We incorporated feedback that the model was vulnerable on healing images, especially those improving from Stage 2 to Stage 1 and from Stage 3 to Stage 2.

### 3.2. Phase 2 Training Results

**Figure 6:**
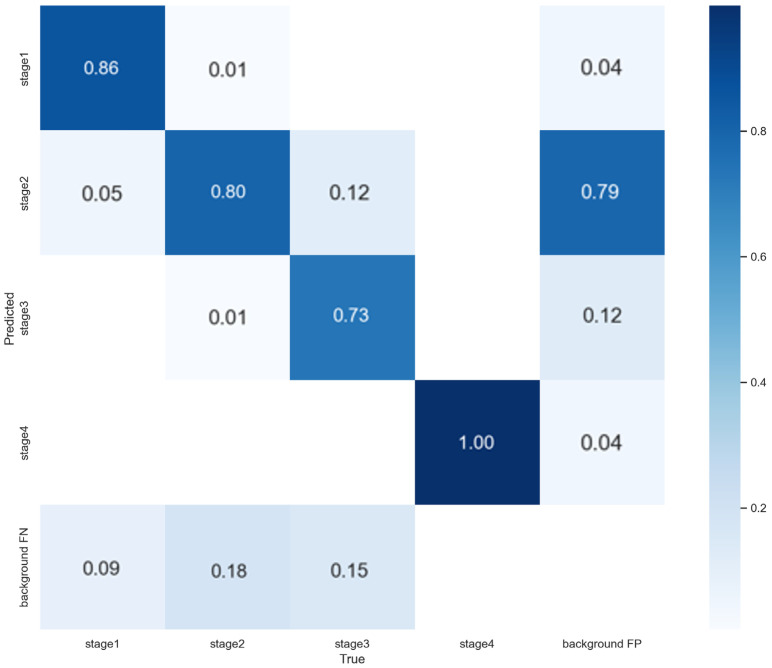
Confusion matrix of the additionally trained model.

After generating an additional 296 images by overpainting fine, spray-like particles and straight lines, we retrained the baseline model with the same hyperparameters for 100 epochs with a batch size of 64. As shown in Figure 6, the per-class accuracies were 0.86 for Stage 1, 0.80 for Stage 2, 0.73 for Stage 3, and 1.00 for Stage 4, and the overall accuracy on the new test set improved from 0.75 to 0.89. These results suggest improved robustness to clinical variability introduced by clinical artifacts. The confusion matrix likewise indicated fewer misclassifications between adjacent stages.

**Figure 7:**
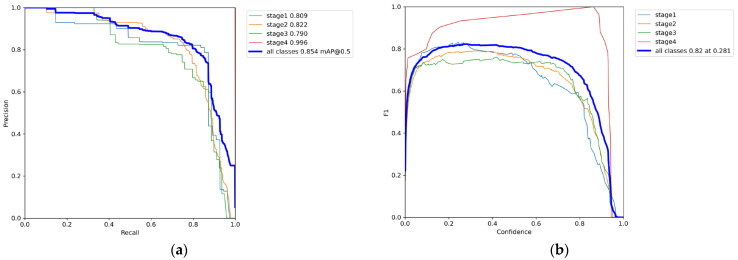

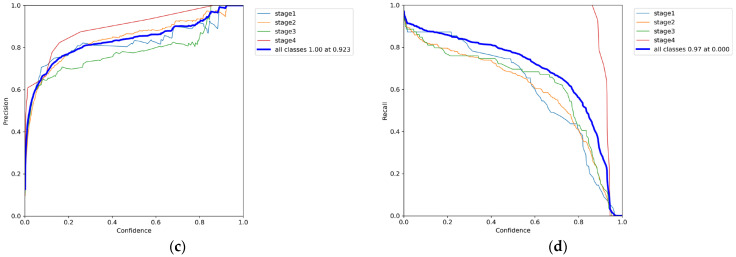
Stage-wise model performance graphs. (**a**) Precision-Recall curve; (**b**) F1 score versus confidence threshold; (**c**) Precision versus confidence threshold; (**d**) Recall versus confidence threshold.

Figure 7 shows the stage-wise performance after the model learned the augmented noise data. We verified correct learning using Precision Recall, F1 confidence, Precision confidence, and Recall confidence.

Figure 7a shows precision versus recall for all classes. The area under each curve gives the class-wise AP, and the overall mAP@0.5 is reported. For Stages 2 and 3, precision declines as recall increases, indicating more false positives in the high-recall region. Figure 7b presents the F1 score as the confidence threshold varies from 0 to 1; the maximum F1 score is 0.82 at a threshold of 0.28, which balances precision and recall. Figure 7c,d plot precision and recall, respectively, as the confidence threshold increases.

### 3.3. 5-Fold Cross-Validation Results

**Figure 8:**
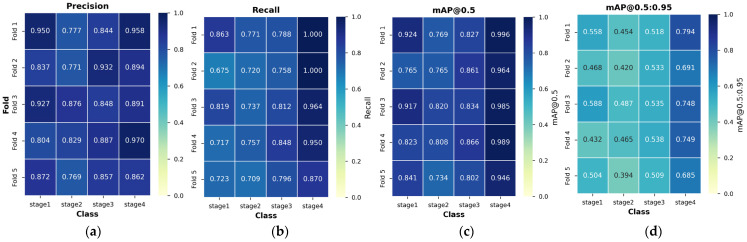
Performance heatmap of 5-fold cross-validation results. Each heatmap shows the distribution of (**a**) Precision, (**b**) Recall, (**c**) mAP@0.5, and (**d**) mAP@0.5:0.95 across five folds and four pressure ulcer stages. Darker blue colors indicate higher performance values. The consistent color patterns across folds demonstrate stable performance, with Stage 4 consistently achieving the highest scores and Stage 2 showing moderate performance across all metrics. The low variation in color intensity within each column indicates minimal fold-to-fold variability for each stage.

To assess the stability and reproducibility of the Phase 2 augmentation strategy, we performed 5-fold cross-validation starting from the Phase 1 best model. The results demonstrated consistent performance across all folds with low variability as follows, Figure 8.

Starting with the best model of Phase 1, each fold was additionally trained for 100 epochs with a batch size of 64 using the Phase 2 augmentation strategy. Overall performance across 5 folds achieved a mean Precision of 86.80% (±1.68%), Recall of 81.38% (±2.97%), mAP@0.5 of 86.20% (±2.28%), and mAP@0.5:0.95 of 55.34% (±2.70%). The standard deviations remained below 3 percentage points for all metrics, indicating stable performance of the Phase 2 augmentation strategy regardless of the specific train-validation split.

Class-wise analysis revealed differential stability patterns across pressure ulcer stages. Stage 4 consistently achieved the highest performance with minimal variability (mAP@0.5: 97.60% ± 1.84%), reflecting both the visual distinctiveness of full-thickness tissue loss and the effectiveness of the saliency-guided augmentation strategy in preserving critical diagnostic features. Stage 2, despite being the most frequent class, exhibited intermediate performance (mAP@0.5: 77.92% ± 3.11%), consistent with the known clinical difficulty in differentiating partial-thickness injuries. Stage 1 showed moderate performance with higher variability (mAP@0.5: 85.40% ± 5.99%), likely due to its subtle visual characteristics and relatively small sample size. Stage 3 demonstrated stable mid-range performance (mAP@0.5: 83.80% ± 2.34%) with the lowest standard deviation in mAP@0.5:0.95 (1.12%), suggesting robust feature learning for full-thickness injuries without extensive tissue loss. Figure 8 illustrates the distribution across folds and stages, with consistently high values in Stage 4 and moderate values in Stage 2–3 across all evaluation metrics. This pattern persisted across all five folds, confirming that the Phase 2 augmentation benefits are not artifacts of a single data split but are consistently reproducible. The low fold-to-fold variability (all standard deviations < 6%) demonstrates that the Phase 2 strategy produces stable improvements over the Phase 1 baseline despite the limited dataset size, validating the robustness of the saliency-guided augmentation approach.

### 3.4. Saliency Map Comparison

Phase 2 lowered the scores on our internal validation set yet raised accuracy on the external set. This pattern indicates that the model learned to ignore bedside nuisance factors that are scarce in the internal data. During Phase 2, we applied overlays that partly covered or altered textures near the lesion without changing the ground-truth label. That training choice promotes robustness but can reduce AP on the comparatively clean internal images. By contrast, the prospectively collected clinical photos often contain dressing borders, residual ointment, specular glare, and atypical viewpoints, so the learned invariance is better matched to clinical deployment. Table 2 presents a side-by-side comparison of Phase 1 and Phase 2 for both datasets. For internal validation, we report accuracy, precision, recall, F1, and mAP. For the external set, we report accuracy.

**Figure 9:**
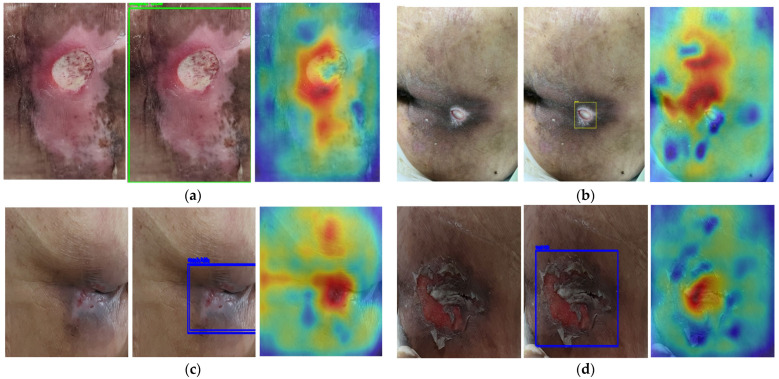
Examples of misclassification in saliency maps.

For Phase 1 training, mAP@0.5 was 0.96 and mAP@0.5:0.95 was 0.68, and accuracy on the 38 hospital images was 0.75. In contrast, for Phase 2 training, mAP@0.5 decreased to 0.85 and mAP@0.5:0.95 decreased to 0.56, whereas accuracy on the same 38 images improved to 0.89. We examined saliency maps for field images misclassified by the Phase 1 model and presented four representative cases in Figure 9. The colors ranging from blue to red indicate an increasing model focus for pressure-ulcer stage determination.

**Figure 10:**
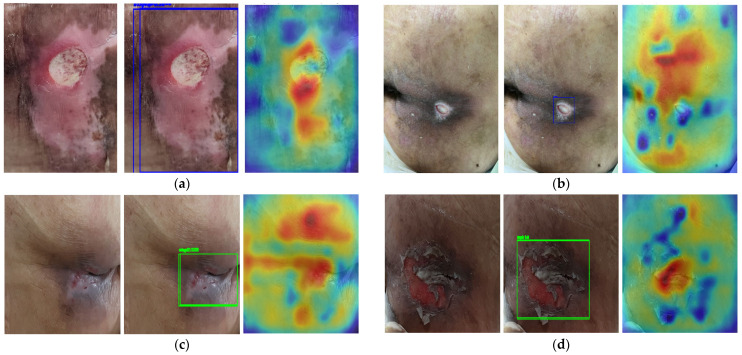
Saliency map examples of the additionally trained model.

In the representative cases, the model localized the lesion but tended to misjudge the stage, focusing mainly on the location when determining the pressure ulcer stage. The correct stages for the misclassified images in Figure 9a–d are Stage 3, Stage 3, Stage 2, and Stage 2, respectively. Therefore, the saliency maps of the model after curriculum learning are shown in Figure 10a–d are Stage 3, Stage 3, Stage 2, and Stage 2. The colors ranging from blue to red indicate an increasing model focus for pressure-ulcer stage determination.

Compared with Figure 9, the saliency maps show that the model considers broader cues to detect the lesion, including attention to relatively white regions in the image. This reflects the fact that real dressings, tape, or other treatment elements are often white. Thus, the noise data created to improve generalization performance is meaningful.

## 4. Discussion

This study improved the clinical generalization of a pressure-ulcer stage classification model. We propose a two-phase curriculum learning procedure. Stage 1 used standard data to establish a stable baseline of discriminative ability. We preprocessed 1282 images for the YOLOv7 detection pipeline and used them for training, thereby establishing a basis for recognizing representative visual cues of the lesion. Stage 2 trained with simulated distractors frequently observed in clinical practice. Examples included indistinct boundaries from healing pressure ulcers, treatment residues, dressing or tape boundaries, and specular highlights and glare introduced during acquisition. These factors are challenging even for clinicians and can confuse models. We generated additional images containing such distractors and designed them to preserve essential lesion cues as much as possible. To this end, we used saliency maps. Because saliency maps show regions the model considers important, we used them to avoid occluding stage-defining regions while adding distractors to surrounding areas. From stages 1 through 3, we randomly selected about 30% of images per stage, created 296 noise images in this manner, and incorporated them into Stage 2 training. The evaluation results support our intent. Internal validation metrics declined slightly overall, but on data that better reflects actual use, such as 38 newly collected hospital images, accuracy rose from 0.75 to 0.89. This indicates enhanced robustness to distribution differences and acquisition noise. Inspection of the confusion matrix showed fewer misclassifications between adjacent stages, specifically between stages 1 and 2 and between stages 2 and 3. In other words, the model became less variable and more consistent in ambiguous boundary cases. Saliency maps before and after training showed that the model no longer relied on a single point or narrow region and instead leveraged broader cues such as relative brightness changes, boundary contours, and texture patterns around the lesion. This demonstrates that training to focus on essential cues despite field distractors working in practice.

Lower internal mAP alongside higher external accuracy indicates a deliberate shift in what the model treats as signal versus nuisance. In Phase 2, label-preserving overlays acted as perturbations that promote invariance to perilesional artifacts; this widens decision boundaries around stage-defining cues and can depress AP on cleaner internal images. The deployment domain differs systematically in illumination, specular highlights, and attachments, so aligning training with these factors improves recognition under domain shift and explains the external gain. Precision-recall analysis shows that the Phase 2 model attains its peak F1 at a lower confidence threshold, indicating a calibration change rather than degraded class separability. Consistently, confusion between adjacent stages decreased, particularly for 1–2 and 2–3. In sum, a modest reduction in internal mAP purchases greater reliability in the target setting.

Comparison with other studies also supports practical deployability. A YOLOv4-based wound detection and stage classification application has previously reported an accuracy of 0.80, while a YOLOv8m-based mobile application for pressure ulcer staging achieved an accuracy of 0.85 [17,20]. In this study, our model achieved an accuracy of 0.89 on an independently collected external test set obtained under consistent clinical conditions. Although differences exist across studies in terms of dataset acquisition protocols, evaluation criteria, and test sample sizes, such numerical comparisons should be interpreted with contextual awareness. Nevertheless, the result provides qualitative support that the proposed model has reached a performance level suitable for practical deployment.

Despite these promising results, this study has several limitations that should be acknowledged. First, the dataset was collected from a single institution, and the external test set was limited to 38 images. Although this set was prospectively collected to mirror bedside conditions, the small scale and single-source nature limit the statistical power and broader generalizability of our findings. Future multi-institutional validation, for which we are preparing under IRB approval, is essential. Second, the clinically informed overlays used in Phase 2 were manually conceived. While effective, this process could introduce unintentional bias, and future work should explore more automated or generative augmentation strategies. Third, our study did not explicitly model all potential sources of clinical variability, such as diverse lighting conditions, camera models, or a wider range of skin tones, which could affect performance. Finally, as noted, our analysis of saliency maps to confirm the model’s improved focus was qualitative. Quantitative analysis would provide more rigorous evidence of the mechanism behind the performance gains.

## 5. Conclusions

Through this procedure, which combines a two-phase curriculum, saliency-guided protection, and targeted injection of clinical noise, we show that accepting a small loss in internal metrics can yield substantial gains in performance and reliability in external environments. The approach applies even in data-limited settings. It readily extends beyond pressure ulcers to related tasks such as wounds and other skin diseases, where background changes, reflections, attachments, and treatment residues are common. While this study demonstrates improved external performance through saliency-guided augmentation, the analysis of saliency maps remains qualitative. Future work should include quantitative metrics such as intersection-over-union between salient regions and annotated lesion boundaries, or attention distribution shift, to provide more rigorous evidence of the model’s improved focus on diagnostically relevant features. We therefore present this procedure as a practical alternative that improves classification consistency in clinical use, reduces the burden of documentation and decision making, and provides a baseline for less experienced clinicians.

## Figures and Tables

**Table 1:** Per-stage distribution of images.

	Stage 1	Stage 2	Stage 3	Stage 4
Total	151	714	319	98
Train	121	571	255	78
Validation	30	143	64	20

**Table 2:** (a) Internal validation metrics. (b) External hospital set (n = 38).

(**a**)					
	mAP@0.5	mAP@0.5:0.95	F1	Precision	Recall
Phase 1	0.96	0.68	0.93	0.94	0.92
Phase 2	0.85	0.56	0.81	0.84	0.86
					
(**b**)					
	Accuracy				
Phase 1	0.75				
Phase 2	0.89				

## Data Availability

The datasets used and/or analysed during the current study are available from the corresponding author on reasonable request.
